# Second General Report of the Trustees and Managers of the Buffalo Hospital of the Sisters of Charity

**Published:** 1852-02

**Authors:** 


### ART. II.—*Second General Report of the Trustees and Managers of the Buffalo Hospital of the Sisters of Charity.*

About one year ago, the managers of "The Buffalo Hospital of the Sisters of Charity" published their first Annual Report. Up to that time, the Institution has received 1,513 patients; the far greater number were on Charity. Since that report, and up to December 1851, 886 patients have been treated in the hospital. Their aggregate stay was 3176 weeks, or an average of three and a half weeks for each. Many were admitted in advanced stages of consumption, or chronic disease, yet only 67 have died. Out-door patients have also received medicine and treatment gratis. Many of the very poor have been clothed, in whole or in part, by the Institution. Of the number, thus lodged, and treated in the hospital, 219 were paid for by the Commissioners of Emigration; 132 were paid for by the Supervisors of Erie County, at about 62 cents per week, a sum which often did not suffice to pay for the medicine, and which never paid half the weekly expense; 23 paid for themselves, in private rooms; 143 paid, in whole or in part, for themselves, in the general ward; 369 charity patients were boarded and treated, gratis, in the hospital; a considerable number more were also charity patients, but not inmates of the house.

There can be no doubt that of about 2,500 sick, treated in the hospital, most of whom are now healthy useful members of society, very many, but for it, would be mouldering in their graves. A distinguished physician, himself a Protestant, says, in his Medical Journal: "The fact that the services of "these intelligent, educated, and pious Sisters, are bestowed without compen- "sation, contributes greatly to the economy of the Institution; but apart from "this, the same capabilities and fidelity, could not be purchased by any pecu- "niary considerations. No salary, however great, could afford a substitute "for motives derived from the religious obligations which urge those devoted "females to consecrate their lives to the Offices of Charity."

In their motherly care of the sick, no distinction of country or religion is permitted. The Sisters have ever strictly adhered to the rule laid down in their first Prospectus. "In the admission of patients no question shall be "made as to what the applicant believes on matters of religion. And when- "ever a patient of any creed may wish to receive spiritual help from the "ministers of his religion, every facility shall be afforded for having his wishes "accomplished."

On this point, the estimable Protestant writer above alluded to, adds:— "It is a Catholic Institution, in the denominational sense of that term; but it "is, at the same time, Catholic in a broader sense than when the term is "applied to designate a particular denomination of Christians. It is, in other "words, in no manner exclusive. It extends relief to the sick of all creeds, "and of no creed, making no discrimination on the score of religious, or non- "religious professions. Patients are always at liberty to elect their own spirit- "ual counselors, and every facility is afforded for the gratification of their "wishes in this respect. We can testify to a degree of delicacy and unob- "trusiveness in this particular, which should satisfy the most fastidious, if not "the most uncharitable. Funeral services, in cases of deaths, are also per- "formed by clergymen of any, and all denominations."

The expenses of such an establishment must necessarily be greater in its beginnings, when alterations, outhouses, repairs, additional furnishing, &c., swell the accounts. The Hospital had, and has debts, but it has not one cent of fixed revenue; it depends entirely upon the Providence of the God of Charity, operating through the generous souls whom He employs in his work. It is then with gratitude to God, and to their benefactors, His honorable stewards, that the Sisters exhibit their accounts, which show less of a deficit than might have been expected:—

| | | | |
|---|---|---|---|
| Paid for medicine, - - | $ 655 23 | Proceeds of Fair, Dec. 1850, | $1,400 00 |
| " provisions, - - - | 3,675 13 | Com. of Emigration, - - | 1,491 35 |
| " fuel, - - - - - | 400 00 | Received from Patients, - | 1,944 72 |
| " clothing nine Sisters and some poor persons, - | 471 50 | Do. from Students, - - - | 305 00 |
| " add'nal furniture, beds, &c. | 592 70 | Do. for their board, - - | 134 00 |
| " repairs, alterations, out-houses, &c. - - | 380 72 | Private donations, - - - | 175 00 |
| " servants' wages, - - | 302 00 | Jenny Lind Concert, - - | 200 00 |
| " insurance, - - - | 50 00 | From Supervisors of the County, | 500 44 |
| " on lots part principal and part interest, - - - | 1,132 74 | From Com. of Emigration. due in last year's report (since pd.) | 340 00 |
| | $7,660 02 | | $6,490 51 |

The above shows a deficit on the year's account of over Eleven Hundred Dollars, and about an equivalent to the amount paid on lots, leaving the amount of indebtedness something less than last year, viz:

| | |
|---|---|
| Amount still due on lots, - - - - - | $2,934 00 |
| Loan of last year, - - - - - - - | 1,000 00 |
| Deficit, supplied by temporary loan, - - - | 1,169 51 |
| | $5,103 51 |

The Sisters have a debt of gratitude to the gentlemen of the Medical Faculty who have most kindly and gratuitously given their services to the institution, in visiting the patients, at least once, every day. During the winter half year, distinguished Professors of the "Buffalo Medical College," Drs. Flint and Hamilton, have spared no pains to comfort and restore to health the sick of the Hospital. During the summer months Drs. Sprague and Winne, and Drs. Burwell and McKay, have shown the kindest zeal in attending the cases of disease and surgery. The house Students also have been most attentive in their departments. To all, the Sisters tender their thanks, hoping that the merciful Saviour will bless them for ministering to Him in his suffering members. To many other friends, who timely helped, or whose kind wishes aided and comforted the Sisters whilst struggling to do good, against the difficulty of beginning such a work, without funds or revenue, they also offer expressions of deepest gratitude, with assurances that their prayers shall ever ascend to implore blessings on the benefactors of the sick and suffering poor.

In their prospectus, on opening their institution, they said: — "The Sis- "ters are ever willing to admit the sick poor gratis, as far as their means will "admit; but as they have no funds, no endowment, they can do but little "at present, except to give gratis their time and kind attentions, hence the "charitable who will send a patient, will be responsible at the following "rates, &c." It will be seen by the present, as well as by the last report, that the Sisters have received a great number gratis, and that they, themselves, have received all the compensation they desire on earth, their simple clothing, their necessary food, and yet, *thanks to God, and to the charitable*, the deficit is not great. With additional buildings and ampler means, they could assuage the sufferings of a still greater number. May the God of Charity send them the means of increasing their usefulness. They repeat now the expressions of their first report. Happy will the Managers and the Sisters be, when some fixed revenue enables them to throw open their doors to all. They respectfully suggest that here, as in Europe, the rich and munificent might found perpetually one or more beds; the founder and his posterity acquiring the right of naming the sick person who will occupy it. Blessed in the records of the future, will be the memory of those who first come forward to spread, till time shall be no more, a bed of comfort and hope for the poor sick sufferer. Their children's children will point with pride to the deed; and when this world shall pass away, the Good Samaritan will, we trust, say to the sick man's friend, "Come blessed of my Father, possess my kingdom, for I was a stranger and you gave me shelter, sick, and you nursed me." Many individuals, touched by this appeal, have expressed a wish to establish a bed, and thus be joint founders of the Hospital: but as yet, nothing of the kind has been done. May God inspire some generous soul to begin, and may others follow that good example. Then, in the future, even more than in the past, we will see the poor sick man tenderly nursed, many saved from the grave, and many, soon restored to health, return to provide for families that otherwise must have been provided for by the county.

"Blessed is he that understandeth concerning the needy and the poor, the Lord will deliver him in the evil day, the Lord preserve him, and give him life, and make him blessed upon the earth, and deliver him not up to the will of his enemies. The Lord will help him on his bed of sorrow: thou hast turned all his couch in his sickness."—Ps. XL.

"He that hath mercy on the poor lendeth to the Lord, and He will repay him."—Prov. XIX.

